# A graft-to strategy of poly(vinylphosphonates) on dopazide-coated gold nanoparticles using *in situ* catalyst activation†

**DOI:** 10.1039/d4ra01116c

**Published:** 2024-03-08

**Authors:** Philipp Weingarten, Sophie R. Thomas, Ana Luiza de Andrade Querino, Kerstin Halama, Moritz Kränzlein, Angela Casini, Bernhard Rieger

**Affiliations:** a WACKER-Chair of Macromolecular Chemistry, Catalysis Research Center, School of Natural Sciences, Department of Chemistry, Technical University of Munich Lichtenbergstraße 4 D-85748 Garching b. München Germany rieger@tum.de; b Chair of Medicinal and Bioinorganic Chemistry, Department of Chemistry, School of Natural Sciences, Technical University of Munich Lichtenbergstraße 4 D-85748 Garching b. München Germany angela.casini@tum.de; c Department of Chemistry, Universidade Federal de Minas Gerais Belo Horizonte MG 31270-901 Brazil

## Abstract

A modular synthetic pathway for poly(diethyl vinylphosphonates) grafting-to gold nanoparticles is presented. Utilising an azide-dopamine derivative as nanoparticle coating agent, alkyne–azide click conditions were used to covalently tether the polymer to gold nanoparticles leading to stable and well distributed colloids for different applications.

## Abstract 2

A graft-to-strategy to tether alkyne-terminated polyesters to the surface of dopazide stabilised AuNPs *via* click chemistry in aqueous conditions *in situ*.

## Graphical abstract

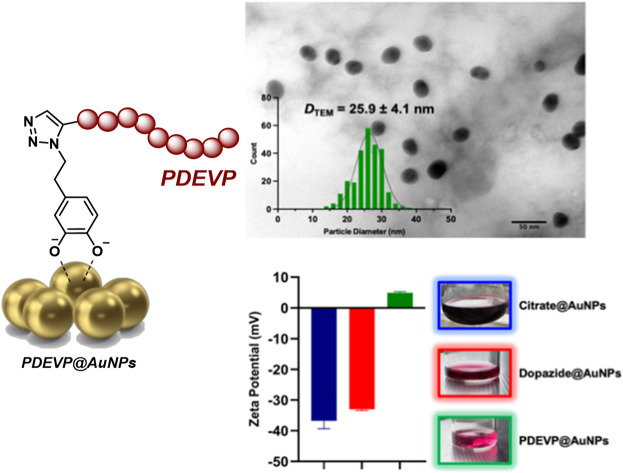

Polymers are materials that have constantly evolved for different applications from the beginning of their discovery until today. One of the most important classes of polymers are functional copolymers, which can be prepared through different methods and feature distinct functionalities, enabling their versatile use.^1^ Rare-earth metal-mediated group-transfer polymerisation (REM-GTP) of polar Michael monomers using yttrium-based catalysts has emerged as a valuable tool for synthesising functional polymers consisting of different microstructures.^2^ Thus, BCPs were carefully tuned to improve their physico-chemical properties, such as lower critical solution temperature (LCST), micelle size or pH responsive behaviour.^3,4^ Additionally, functional side-chains (allyl groups) and end-groups enhanced the scope of application for these polymers by facilitating the attachment of ionic moieties or biologically active molecules.^5^ Interestingly, non-toxic poly(diethyl vinylphosphonate) (PDEVP)-containing block copolymers (BCPs) hold promise as candidates for encapsulation and delivery of anti-cancer agents.^4,6,7^ Functional side-chains of such polymers could also enhance their drug delivery potential by creating crosslinked nanoparticles.^6^

To date, poly(vinyl phosphonate)-based polymers could be successfully applied for surface coatings in a few studies. For example, flame retardant properties were utilised as macroscopic polymer films on polycarbonates showing good performance due to their high thermal stability and morphological behaviour upon flame treatment.^8^ On a molecular scale, a grafting-from strategy including methyl methacrylate and DEVP was successfully developed to obtain polymer brushes on silicon surfaces with temperature and pH-responsive characteristics that might serve as proton conducting films or scaffolds for cell growth.^9^ Moreover, azide end-group functionalised PDEVP have been utilised to immobilise the polymer on multi-walled carbon nanotubes.^10^

To the best of our knowledge, no other poly(vinyl phosphonate)-based surface coating applications are known. However, coatings with functional poly(vinyl phosphonates) could be very relevant for different applications of nanomaterials. Thus, in search of anchoring groups for the modification of surfaces with functional PDEVP, dopamine appeared as a popular choice, specifically for grafting-to methods.^11^ The catechol moiety of polydopamine (PDA) offers strong binding on an array of different material surfaces, including superhydrophobic surfaces.^12^ For proof-of-concept experiments, gold nanoparticles (AuNPs) were selected as the surface material due to their advantageous properties, such as high surface area to volume ratio, established characterisation methods, and interesting electronic and physical properties.^13^ Moreover, examples of PDA-coated gold nanoparticles (PDA@AuNPs), synthesised by self-polymerisation of dopamine on the gold surfaces, are already reported to have well controlled Au core size and PDA coating layer thickness.^14,15^ Thus, PDA@AuNPs have been used in various applications, most commonly as therapeutic^15,16^ and sensing agents,^17^ with some reports of their use in catalysis^18^ and energy^19^ conversion.

Therefore, we report here the synthesis of alkyne-terminated poly(vinyl phosphonates) *via in situ* catalyst activation and a facile method to graft these polymers to the surface of plasmonic dopazide-stabilised AuNPs *via* click chemistry in aqueous conditions. Taking inspiration from our previous work,^20^*in situ* σ-bond activation of [Cp_2_Y(CH_2_TMS)(THF)] (Y, TMS = trimethylsilyl, THF = tetrahydrofuran) using an alkyne-containing *sym*-collidine derivative was used to achieve alkyne-terminated polymers (Scheme 1 and S1†). The synthetic route was extended to different types of Michael monomers (DEVP, 2-vinyl pyridine (2VP), iso-propenyl oxazoline (iPOx), and a BCP consisting of DEVP and diallyl vinylphosphonate (DAlVP)) (Scheme S2†). Successful catalyst activation, end-group functionalisation, and polymerisation was demonstrated by electrospray ionisation mass spectrometry (ESI-MS), size exclusion chromatography (SEC), and ^1^H and ^1^H DOSY NMR spectroscopy on the purified polymers (Fig. S1–S16 and Table S1†). Apart from temperature responsive PiPOx and hydrophobic and pH responsive P2VP, PDEVP offers hydrophilicity, temperature responsiveness and a high degree of biocompatibility,^21^ and therefore was selected for anchoring to AuNPs. To tether the functional PDEVP to the dopamine coated AuNPs, a dopamine derivative was selected,^22^ and its amino group replaced with an azido moiety *via* a diazo-transfer,^23^ providing a robust functional group for popular click reactions (Scheme 1, see ESI for details†).

**Scheme 1:**
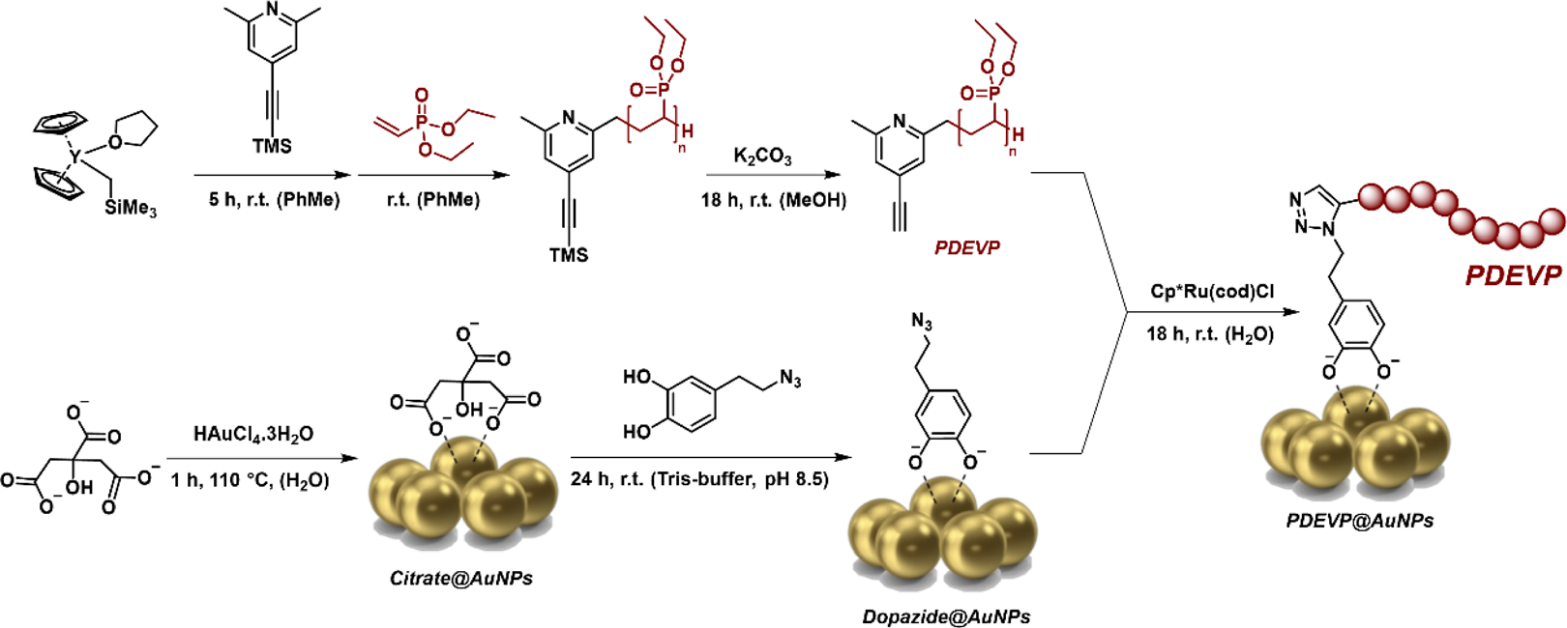
Synthetic scheme for the synthesis of PDEVP *via in situ* catalyst activation (top) and dopazide@AuNPs formed *via* a ligand exchange reaction (bottom), which are then combined *via* a RuAAC reaction to produce PDEVP@AuNPs.

To demonstrate the presence of the viable alkyne as an end-group, a qualitative study was performed utilising ruthenium-catalysed azide–alkyne click (RuAAC) conditions to react the alkyne with the fluorescent compound 3-azido-7-hydroxycoumarin (see ESI for details, Fig. S17†). Photoluminescence studies of the obtained polymer showed fluorescence at the expected wavelength (Fig. S18†). These reaction conditions were the basis for the decoration of dopazide-coated AuNPs, which is the first step to creating hybrid materials consisting of PDEVP and AuNPs.

Before the click reaction can be performed, the dopazide ligand must first be coated onto the AuNP surface *via* a ligand exchange reaction with citrate stabilised AuNPs (citrate@AuNPs) to achieve dopazide@AuNPs (Scheme 1).^24^ The water-soluble ruby-red dopazide@AuNPs were then purified by centrifugation to remove the excess of free dopazide and citrate. The final step to conjugate the alkyne terminated polymer to the dopazide@AuNPs was performed *via* a click reaction with the Cp*RuCl(cod) catalyst using similar conditions as described above (Scheme 1, see ESI for details†).

The characterisation of the PDEVP@AuNPs was then attained *via* different methods and directly compared to the precursor AuNPs (dopazide@AuNPs and citrate@AuNPs), as summarised in Table 1. UV-visible absorption spectroscopy was first used to evaluate the presence of AuNPs due to the typical surface plasmon resonance (SPR) band at 520–530 nm (Fig. 1A), indicative of spherical NPs with an average size of *ca*. 20 nm.^25^ The PDEVP@AuNPs feature a SPR band around 536 nm, comparable to the band of the dopazide@AuNPs and citrate@AuNPs (Fig. 1A). Furthermore, a broad peak around 270 nm appears that can be attributed to the presence of the polymer; slightly blue-shifted compared to the intense π–π* intraligand transition of the polymer alone (*ca.* 290 nm) and of the dopazide@AuNPs (*ca*. 280 nm) (Fig. S19†).

**Table 1:** Summary of the key characterisation results for the PDEVP@AuNPs and their precursors (citrate@AuNPs and dopazide@AuNPs), as well as for the two control experiments (test (i) and test (ii))

Sample	*D*_TEM_ [nm]	*D*_h_ [nm]	*ζ*-Potential [mV]
Citrate@AuNPs	25.8 ± 7.1	32 ± 0.9	−38.2 ± 2.5
Dopazide@AuNPs	27.8 ± 7.5	48 ± 0.5	−31.5 ± 1.0
PDEVP@AuNPs	25.9 ± 4.1	78.8 ± 1.5	+4.9 ± 0.4
Test (i) (polymer w/o alkyne)	—	122.9 ± 9.8	+3.1 ± 0.3
Test (ii) (no cat.)	—	70.0 ± 2.0	−6.7 ± 0.4

**Fig. 1:**
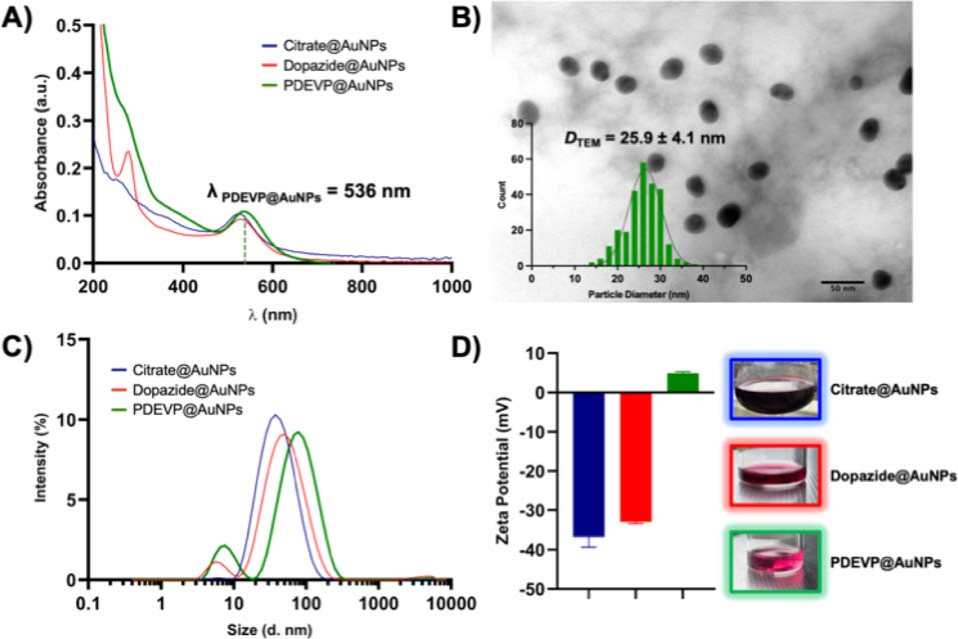
Characterization of citrate@AuNPs, dopazide@AuNPs and PDEVP@AuNPs *via* different methods. (A) UV-vis absorption spectra in Milli-Q water. (B) Representative TEM image (scale bar 50 nm) of PDEVP@AuNPs with particle size histogram displaying an average size of 25.9 ± 4.1 nm. Representative plots of (C) intensity size distribution by DLS, and (D) zeta potential of nanoparticles in Milli-Q water displaying the average values from 3 repeats (insert: images of each NP solution).

The average particle size and size distribution of the PDEVP@AuNPs was next evaluated by transmission electron microscopy (TEM) (Table 1). A representative TEM image is reported in Fig. 1B, showing the well-distributed monodisperse quasi-spherical PDEVP@AuNPs with an average diameter (*D*_TEM_) of 25.9 ± 4.1 nm, which is comparable to the precursor AuNPs (*D*_TEM_ dopazide@AuNPs = 27.8 ± 7.5 nm and *D*_TEM_ citrate@AuNPs = 25.8 ± 7.1 nm, Fig. S20, S21,† and Table 1) and in line with literature values.^26^ Additionally, the TEM images showed no apparent aggregation of the AuNPs, highlighting their stability throughout the reaction steps. Furthermore, dynamic light scattering (DLS) can be used to measure both the core NP size and the size of the surface coating of the AuNPs in solution. *Via* this method, analysis of an aqueous solution of the PDEVP@AuNPs revealed an average hydrodynamic diameter (*D*_h_) of 78.8 ± 1.5 (Fig. 1C and Table 1), suggesting a polymer shell-AuNP structure. This is also apparent, albeit to a lesser extent, in the dopazide@AuNPs and citrate@AuNPs with smaller *D*_h_ values of 48 ± 0.5 and 32 ± 0.9 nm, respectively (Fig. 1C and Table 1), due to the shorter linker and stabiliser sizes. The presence of the polymer clicked to the dopazide@AuNPs was further confirmed by measuring the surface *ζ*-potential of the different NP solutions (Fig. 1D and Table 1). An aqueous PDEVP@AuNP solution showed a small *ζ*-potential of +4.9 ± 0.4 mV, as expected from a neutral polymer shell. This is at variance to the precursor AuNPs featuring negative *ζ*-potentials (Fig. 1D and Table 1), as also seen for similar systems in the literature.^27^

Fourier transform infrared (FTIR) spectra showed the loss of the azide stretch at *ca*. 2000 cm^−1^ from the dopazide ligand after the click reaction to form the PDEVP@AuNPs (Fig. S22†). Furthermore, to estimate the degree of ligand functionalisation on the PDEVP@AuNPs, thermogravimetric analysis (TGA) was performed. From the TGA-profile (Fig. S23†), one broad event is observed starting at *ca*. 100 °C until 472 °C, with a total weight loss of approximately 30 wt%, likely to be attributed to the loss of the organic bound ligands.

To rule out the possibility that the polymer was merely adsorbed to the NP surface, a control experiment was performed using the same reaction conditions as the title reaction, but featuring a structurally similar polymer without the alkyne moiety (test (i)).^20^ Upon reaction completion, a colour change of the solution was observed, from red to pale pink, and the solution became slightly turbid, revealing the presence of suspended particles. The results of the characterisation are summarised in Table 1 (test i). The UV-vis absorption spectrum of test (i) appeared comparable to that of the PDEVP@AuNPs with an SPR band at 536 nm and a broad peak in the UV region at *ca*. 260–270 nm (Fig. S24†); the latter being attributable to polymer coating the NP surfaces. The presence of suspended/aggregated NPs was further evidenced by the DLS measurement, with test (i) displaying a *D*_h_ of 122.9 ± 9.8 nm, significantly larger than the PDEVP@AuNPs (Table 1 and Fig. S25A†). The *ζ*-potential was slightly positive, similar to what was observed for the PDEVP@AuNPs (Fig. S25B†). Furthermore, the IR spectrum showed a significant concentration of the dopazide moiety in the supernatant (Fig. S26†), in line with the observed colour change and turbidity of the solution upon polymer addition.

A second control reaction was performed using the dopazide@AuNPs and the polymer with an alkyne moiety, in absence of the catalyst (test ii). Interestingly, even without the catalyst, the click reaction can occur, albeit to a lower extent.^28^ Nevertheless, the incomplete click reaction can be observed in the UV-vis spectrum in Fig. S27,† featuring two absorption bands for test ii at 230 and 280 nm, likely corresponding to a combination of the PDEVP polymer alone, dopazide@AuNPs and PDEVP@AuNPs. The DLS and *ζ*-potential results (Table 1) further support this hypothesis (Fig. S28A and B,† respectively). Additionally, the FTIR spectrum of test ii still displays the azide peak at *ca*. 2000 cm^−1^, as expected due to the reaction only occurring partially (Fig. S29†). Overall, these results point towards the successful engrafting of the polymer on the AuNP surfaces *via* click chemistry to provide a well-defined nanostructure.

In summary, we report here a straightforward protocol for grafting alkyne terminated polyesters to the surface of plasmonic dopazide-stabilised AuNPs *via* click chemistry in aqueous conditions *in situ*. Comprehensive characterisation of the PDEVP@AuNPs and its precursors was performed by different methods to confirm the successful click reaction, demonstrating the formation of single polymeric specie, as well as the stability of the NPs during the multiple steps. Combining the tuneability of the BCPs' properties with the herein reported alkyne at the polymer's end-group means that precise modification under mild conditions is possible. Thus, the accessibility to orthogonal functionalisation of surfaces, proteins, and biologically relevant peptides or drugs with BCPs is enhanced. More specifically, PDEVP@AuNPs are interesting for different applications, such as the obtainment of nanosensors *via* the ordered deposition of nanoparticles on nanofibers,^29^ or other highly curved surfaces, as well as for applications in continuous flow catalysis or lithography on silicon substrates.^30^ Further, the possibility to stably coat surfaces with BCPs is interesting to confer different properties to the material depending on the type of monomers and their functionalisation. From the perspective of the AuNPs for biomedical applications, such as drug delivery, imaging or for the photothermal therapy of tumours,^31^ colloids featuring a dense brush polymer surface can display high stability and tuneable pharmacokinetic properties. In general, polymeric ligands improve the long-term stability of AuNPs and increase hydrophilicity of the surface. In addition, AuNPs size can be adjusted with polymer coating. Finally, BCPs could represent a new class of antifouling coatings to render NPs more biocompatible.^32^ All these features can be rationally implemented in AuNPs exploiting BCPs' highly versatile combinatorial properties, to achieve finely engineered nanomaterials.

## Conflicts of interest

There are no conflict of interest to declare.

## Supplementary Material

RA-014-D4RA01116C-s001
