# Supplementary material for: A graft-to strategy of poly(vinylphosphonates) on dopazide-coated gold nanoparticles using in situ catalyst activation
Source: RSC Adv. 2024 Mar 8;14(12):8145–9. doi: 10.1039/d4ra01116c (PMC10921843; doi:10.1039/d4ra01116c)
Supplement: RA-014-D4RA01116C-s001 [file RA-014-D4RA01116C-s001.pdf]

Electronic Supplementary Material (ESI) for RSC Advances.
This journal is © The Royal Society of Chemistry 2024

A graft-to strategy of poly(vinylphosphonates) on dopazide-coated gold nanoparticles using *in situ* catalyst activation

Philipp Weingarten,[‡,a] Sophie R. Thomas,[‡,b] Ana Luiza de Andrade Querino,[‡,b,c] Kerstin Halama,[a] Moritz Kränzlein,[a] Angela Casini,*[,b] Bernhard Rieger*[,a]

[a] *WACKER-Chair of Macromolecular Chemistry, Catalysis Research Center, School of Natural Sciences, Technical University of Munich, Lichtenbergstraße 4, D-85748 Garching b. München, Germany.*

[b] *Chair of Medicinal and Bioinorganic Chemistry, School of Natural Sciences, Technical University of Munich, Lichtenbergstraße 4, D-85748 Garching b. München, Germany.*

[c] *Department of Chemistry, Universidade Federal de Minas Gerais, Belo Horizonte, MG 31270-901, Brazil.*

[‡] These authors contributed equally to this manuscript.

Corresponding Authors

* angela.casini@tum.de; rieger@tum.de

1. Materials and methods

General Information

All reactions were carried out under argon atmosphere using standard Schlenk techniques. All glassware was heat dried under vacuum prior to use. Polymerisations with moisture and air-sensitive reactants were carried out in a MBraun LabMaster120 glovebox filled with argon 4.6 from Westfalen or using standard Schlenk techniques. All glassware was heat-dried prior to use. All chemicals were purchased from Sigma-Aldrich, ABCR, or TCI Europe and used without further purification unless otherwise stated. Dichloromethane, tetrahydrofuran, toluene, and pentane were dried using an MBraun SPS-800 solvent purification system and stored over 3 Å molecular sieve. Diethyl vinylphosphonate (DEVP) and diallyl vinylphosphonate (DAlVP) were synthesised according to literature procedures.[1] 2-vinylpyridine (2VP), *iso*-propenyl oxazoline (*i*POx), DEVP and DAlVP were dried over CaH_2 for several days and distilled prior to use. $LiCH_2TMS$,[2] $Cp_2YCH_2TMS(thf)$,[3] 4-chloro-2,6-dimethylpyridine,[4] PDEVP polymer without terminal alkyne[5] and the citrate stabilised gold nanoparticles[6] were prepared according to literature procedures.

NMR Spectroscopy

NMR spectra were recorded on a Bruker AV-400HD spectrometer. ^{1}H (400 MHz), ^{13}C (125 MHz) and ^{31}P (162 MHz). NMR spectroscopical shifts δ were reported in ppm relative to the residual proton or carbon signal of the deuterated solvent. Deuterated solvents ($CDCl_3$, benzene-d_6) were purchased from Sigma-Aldrich or Deutero and dried over 3 Å molecular sieves prior to use.

Size-exclusion chromatography

Molecular weights and polydispersity of PDEVP, PiPOx, and PDEVP-*b*-PDAlVP (c = 2.5 mg mL^{-1}) were determined by SEC-MALS using a Wyatt Dawn Heleos II MALS light scattering unit and a Wyatt Optilab rEX 536 RI unit in THF:H_2O = 1:1 (with 9 g/L *tetra*-*n*-butyl-ammonium bromide and 272 mg/L 2,6-di-*tert*-butyl-4-methylphenol added) as eluent at 40 °C on two Agilent PolarGel-M columns; for absolute molecular weight (triple detection) determination of different polymers, the refractive index increments were used: PDEVP dn/dc = 0.0922 mL g^{-1}, PiPOx dn/dc = 0.1225 mL g^{-1}.[7]

Average absolute molecular weights and polydispersities of P2VP was determined via size-exclusion chromatography with a sample concentration of 2.5 mg mL^{-1}. Measurements were performed on an Agilent PL-GPC 50 (Santa Clara, CA, USA) with an integrated RI unit, two light scattering detectors (15° and 90°), and a differential pressure viscosimeter with two Agilent PolarGel M columns. As eluent N,N-dimethylformamide (with 2.096 g/L lithium bromide added) at 30 °C was used. Absolute molecular weights of P2VP were determined using dn/dc = 0.149 mL g^{-1} from literature.[7]

Dialysis

Purification of products via dialysis was performed with *Spectra/Por 1* dialysis membranes (regenerated cellulose) from *Spectrum*™ against deionised water. The molecular weight cut-off (MWCO) of the membranes was 6-8 kDa and 3.3 mL cm^{-1} volume-length ratio was used. The PDEVP@AuNPs were purified with cellulose dialysis membranes (Sigma-Aldrich, D9652) with a MWCO of 14 kDa.

Lyophilisation

The polymer samples subject to freeze-drying were dissolved in either ultrapure water or 1,4-dioxane and frozen under constant rotation in liquid nitrogen. For lyophilisation, a VaCo 5-II-D from Zirbus Technology GmbH was used, the pressure was adjusted to 2 mbar with a condenser temperature of -90 °C.

Electronspray Ionisation Mass Spectrometry

ESI-MS was measured using a Thermo Fisher Scientific Exactive Plus Orbitrap in positive mode in HPLC acetonitrile straight from the reaction mixture without quenching.

Transmission electron microscopy

Transmission electron microscopy (TEM) measurements were performed on a JEOL JEM 1400 plus instrument at an acceleration voltage of 120 kV. Before analysis, the samples were suspended in bidistilled water and dispersed ultrasonically before being deposited on a carbon-coated copper mesh. TEM analysis of the AuNPs (citrate@AuNPs, dopazide@AuNPs and PDEVP@AuNPs) was carried out using the same equipment and acceleration voltage after deposition of an aqueous solution of the AuNPs (in Milli-Q water) on 300 mesh copper grids coated with holey carbon film.

Thermogravimetric Analysis (TGA)

TG measurements were performed on a TG Q5000 (TA Instruments) from 25 °C to 800 °C under argon with a stream of 25 mL min^{-1}.

Dynamic light scattering (DLS) and Zeta Potential Measurements

The average hydrodynamic particle size and zeta potential measurements were acquired using the Zetasizer Nano apparatus (Malvern Instrument Ltd., Malvern).

Infrared (IR) spectroscopy

IR spectra were recorded on a Frontier™ Fourier transform (FT) IR spectrometer from PerkinElmer.

UV-Visible spectroscopy

UV-vis spectra were recorded on a Cary 60 UV-Vis spectrometer from Agilent Technologies.

Fluorescence spectroscopy

Fluorescence spectroscopy was performed on a Jasco (Jasco FP-8300, Spectra Manager software 2.13). The concentration of polymers in methanol was determined to be 1.0 mg/mL. Corresponding substrates were prepared in 0.5 mM solutions. Fluorescence spectroscopy was performed on a Jasco (Jasco FP-8300, Spectra Manager software 2.13).

2. Syntheses

4-Iodo-2,6-dimethylpyridine

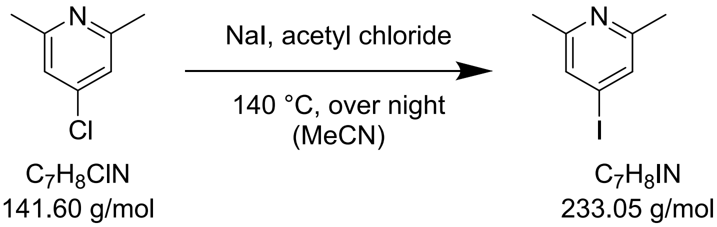

In an autoclave, 3.00 g (21.2 mmol, 1.0 eq.) 4-chloro-2,6-dimethylpyridine and 19.1 g (127 mmol, 6.0 eq.) sodium iodide were suspended in 50.0 mL acetonitrile. 2.27 mL (2.49 mg, 31.8 mmol, 1.5 eq.) acetyl chloride was added dropwise and the reaction mixture was stirred over night at 140 °C. Afterwards, aqueous solutions of K_2CO_3 (25.0 mL, 10.0 wt%), Na_2SO_3 (25.0 mL, 5.00 wt%) and $Na_2S_2O_3$ (20.0 mL, concentrated solution) were added to the cold suspension. Ethyl acetate was added until phase separation could be observed. After extracting the aqueous phase with ethyl acetate twice, the combined organic phases were dried over Na_2SO_4 and the solvent removed in vacuo. Purification via sublimation with dry ice cooling (33.0 °C, $p = 0.1 \times 10^{-2}$ mbar) yielded 2.26 g (9.69 mmol, 45%) 4-iodo-2,6-dimethylpyridine as a white solid.

^{1}H-NMR (500 MHz, $CDCl_3$, 300 K): δ (ppm) = 7.38 (s, 2H, H_{Ar}), 2.47 (s, 6H, CH_3).

^{13}C-NMR (101 MHz, $CDCl_3$, 300 K): δ (ppm) = 159.0 (s), 129.9 (s), 106.8 (s), 24.4 (s).

EA: calculated: C 36.08%, H 3.46%, N 6.01%, I 54.45%.

found: C 36.27%, H 3.33%, N 6.07%, I 54.04%.

The analytical data match those reported in the literature.[4]

2,6-Dimethyl-4-((Trimethylsilyl)ethynyl)pyridine

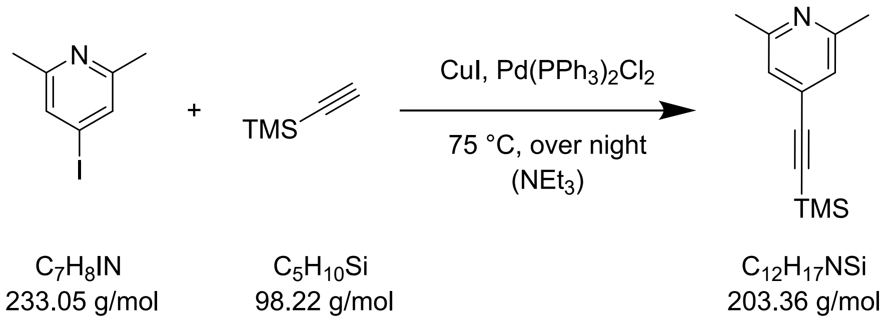

1.99 g (8.53 mmol, 1.00 eq.) 4-Iodo-2,6-dimethylpyridine and 120 mg (16.3 mmol, 0.02 eq.) bis(tri-phenylphosphine) palladium(II) dichloride were dissolved in 150 mL triethylamine. After adding 1.47 mL (1.17 mg, 11.9 mmol, 1.40 eq.) trimethylsilylacetylene and 16.3 mg (85.3 mmol, 0.01 eq.) copper (I) iodide, the reaction mixture was stirred at 75 °C over night. The solid residue was filtered off before the solvent was removed in vacuo. Purification via column chromatography (H/Net_3 = 99:1, aluminium oxide as stationary phase) yielded 1.16 g (5.70 mmol, 67%) 2,6-dimethyl-4-((trimethylsilyl)ethynyl)pyridine.

^{1}H-NMR (400 MHz, C_6D_6, 300 K): δ (ppm) = 6.84 (s, 2H, H_{Ar}), 2.29 (s, 6H, CH_3), 0.24 (s, 9H, Si($CH_{3)3}$).

ESI-MS: calculated: 204.1209 $[M–H]^+$, found: 204.1206 $[M–H]^+$.

EA: calculated: C 69.92%, H 8.53%, N 6.89%, I 13.81%.

found: C 70.68%, H 8.43%, N 6.99%, I 13.70%.

The analytical data match those reported in the literature.[8]

4-(2-Azidoethyl)benzene-1,2-diol

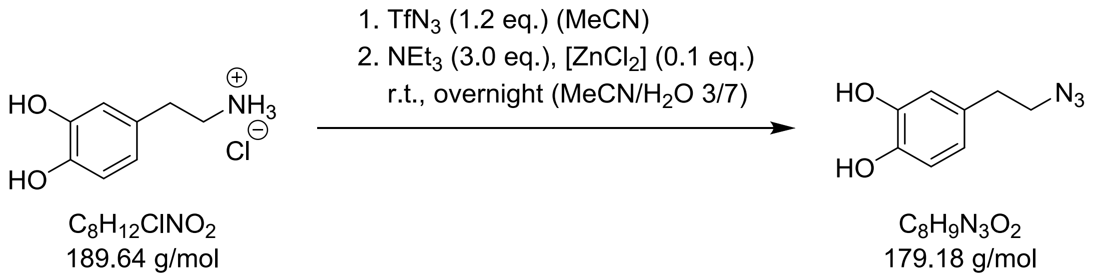

NaN_3 (3.09 g, 47.5 mmol, 3.0 eq.) was suspended in 30 mL MeCN, cooled to 0 °C and 3.19 mL Tf_2O (5.36 g, 19.0 mmol, 1.2 eq.) were added slowly. After 2 h, the *in situ* formed TfN_3 was slowly added to a solution of 3.00 g dopamine · HCl (15.8 mmol, 1.0 eq.), 215 mg $ZnCl_2$ (1.58 mmol, 0.1 eq.), and 6.58 mL triethylamine (4.80 g, 47.5 mmol, 3.0 eq.) in 75 mL MeCN/H_2O (7/3) at room temperature and the reaction mixture was stirred overnight. MeCN was removed in vacuo, 200 mL water were added to the reaction mixture and the aqueous phase was extracted with EtOAc (3 x 250 mL). The combined organic layers were dried over Na_2SO_4, filtered and the solvent was removed in vacuo. After column chromatography (silica, CH_2Cl_2/MeOH 95/5), the product was obtained as a dark brown oil (2.50 g, 14.0 mmol, 88%).
^{1}H-NMR (400 MHz, $CDCl_3$, 300 K): δ (ppm) = 6.79 (d, ^3J = 8.1 Hz, 1 H, H_{Ar}), 6.73 (d, ^4J = 2.1 Hz, 1 H, H_{Ar}), 6.64 (dd, ^3J = 8.1 Hz, ^4J = 2.1 Hz, 1 H, H_{Ar}), 3.45 (t, ^3J = 7.2 Hz, 2 H, CH_2), 2.77 (t, ^3J = 7.2 Hz, 2 H, CH_2).

The analytical data match those reported in the literature.[9]

***in-situ* CH-bond activation of $Cp_2Y(CH_2TMS)(thf)$**

The *in-situ* CH-bond activation of the methyl pyridine compound is monitored via kinetic ^{1}H-NMR experiment in a J-Young NMR tube. 5.11 mg (0.0135 mmol, 1.0 eq.) $Cp_2YCH_2TMS(thf)$ are dissolved in 0.3 mL dry benzene-d_6. The solution is added to 3.29 mg (0.0162 mmol, 1.2 eq.) 2,6-dimethyl-4-((trimethylsilyl)ethynyl)pyridine in 0.3 mL dry benzene-d_6 at room temperature. ^{1}H-NMR spectra are measured immediately after addition (5 minutes), every 15 minutes for one hour and finally every hour for 5 hours.

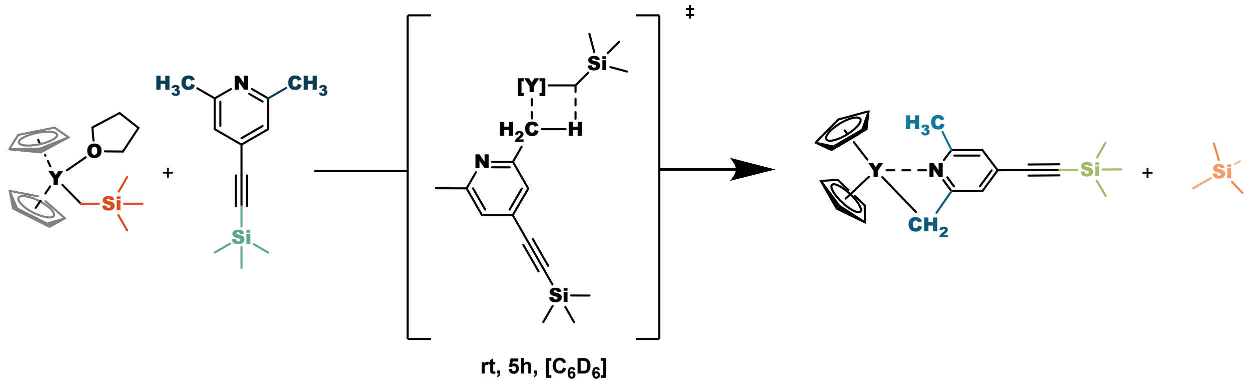

Scheme S1: In situ CH-bond activation of Cp2Y(CH2TMS)(thf) using the pyridine alkyne derivative.

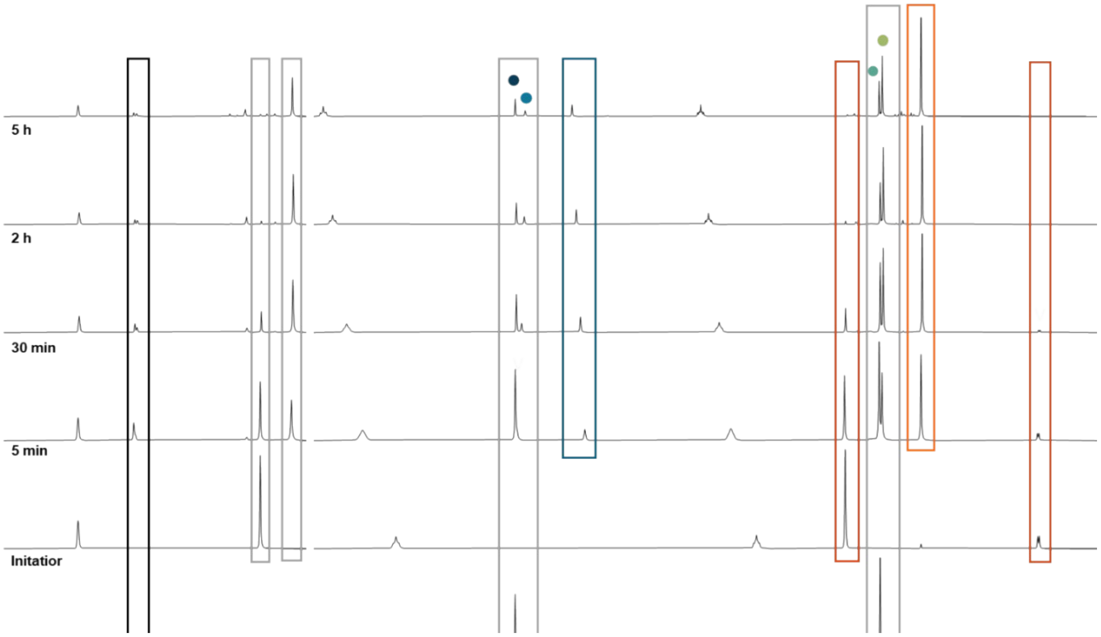

Figure S1:^{1}H-NMR spectrum of CH-bond activation. Full conversion is observed after 5 h by the appearing signal of the forming CH_2 group (dark blue) and the vanishing signal of Y-<u>CH_2</u>TMS (dark red, –0.6 ppm).

3. Polymer synthesis and functionalisation

Group-transfer polymerisation of Michael monomers

After full conversion of the CH-bond activation, the respective equivalents of Michael monomer were added in one portion. One aliquot (0.1 mL) was taken and subsequently quenched by addition of 0.4 mL wet CD_3OD (calculation of conversion via ^{1}H-NMR spectroscopy). After full conversion was observed, the reaction mixture of the homopolymerization was then quenched by the addition of 0.5 mL EtOH and subsequently precipitated from pentane (50 mL). In terms of the block copolymerization (Table S1, entry 7), the respective amount of DAlVP was added in one portion. One aliquot (0.1 mL) was taken and subsequently quenched by addition of 0.4 mL wet CD_3OD (calculation of conversion via ^{1}H-NMR spectroscopy). After full conversion was observed, the reaction mixture of the block copolymerization was then quenched by the addition of 0.5 mL EtOH and subsequently precipitated from pentane (50 mL). After centrifugation, the liquid phase was decanted off. The polymer was dissolved in 1,4-dioxane and lyophilised overnight. The product was obtained as a white solid. Each aliquot was used for SEC analysis of the containing polymer species.

Table S1: Group-transfer polymerization results with *in-situ* activated $Cp_2Y(PyCCTMS)$.

Monomer	[M]:[Y]^a [mol]:[mol]	$M_{n,theo}$^{b} [kg/mol]	$M_{n,abs}$^{c} [kg/mol]	$Đ^c$ [-]	I.E.^{d} [%]
DEVP	50:1	8.7	19.6	1.09	44
DEVP	100:1	16.7	31.5	1.09	53
DEVP	200:1	33.3	58.8	1.11	57
DEVP	400:1	66.8	113	1.10	59
2VP	100:1	10.0	17.8^e	1.03	60
iPOx	100:1	10.8	20.8	1.53	52
DEVP-*b*-DAlVP^f	100:	15.1 (A)	26.0 (A)	1.13	58 (A)

10:1	(AB)

[a]Catalyst to monomer ratio. [b]Theoretical molecular weight *via* $M_{n,theo} = M_{DEVP} \times X_{DEVP} \times ([Monomer]/[Y])$. [c]Absolute molecular weight and polydispersity determined *via* SEC-MALS (THF:H_2O = 1:1) (Figures S3 to S9). [d]Initiator efficiency as I.E. = $M_{n,theo}/M_{n,abs} \times 100\%$. [e]Absolute molecular weight and polydispersity determined *via* SEC in DMF. [f]Polymerization *via* sequential addition of monomers. Molecular weight and I.E. given for block A, polydispersity for the AB BCP.

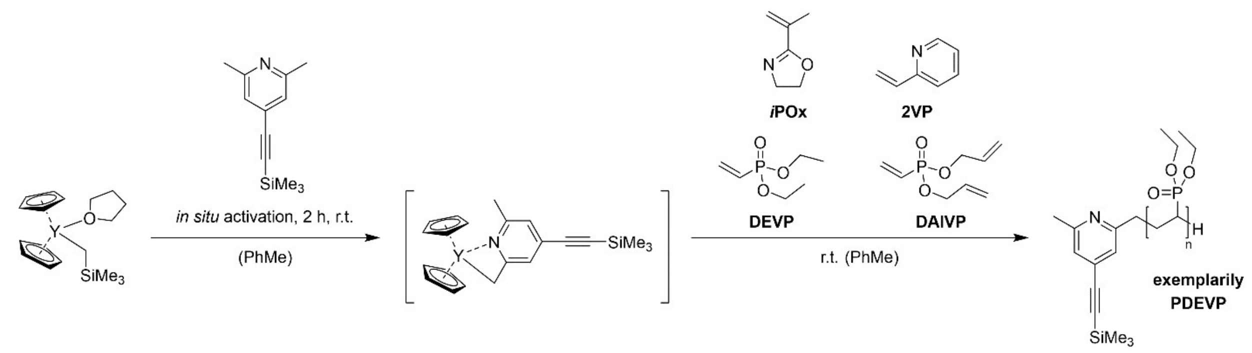

Scheme S2: *In-situ* activation of $Cp_2YCH2TMS(thf)$ with pyridine alkyne and subsequent polymerisation.

TMS deprotection procedure of polymers from group-transfer polymerisation

Alkyne functionalised PDEVP (200 mg, 1.0 eq.) was dissolved in 12 mL MeOH, the calculated amount of K_2CO_3 (20 eq.) was suspended, and the obtained suspension was stirred overnight. After removal of the solvent, the mixture was dissolved in water and dialysed for 24 h (MWCO 6-8 kDa). Lyophilisation afforded the deprotected polymer.

Functionalisation of polymers with Coumarine-N_3 using azide-alkyne click reaction

Deprotected PDEVP (50.0 mg, 1.0 eq.) was dissolved in 3 mL water, 1 mL of a solution of 3-azido-7-coumarine (10 eq., 2.17 µM in water) was added, and 0.1 mL of a solution of Cp*Ru(cod)Cl (0.1 eq., 0.002 µM in water) was added. The obtained reaction mixture was stirred overnight and subsequently dialysed (MWCO 6-8 kDa). Lyophilisation afforded the polymer as a yellowish solid.

Preparation of the Dopazide functionalised AuNPs *via* ligand-exchange

The dopazide@AuNPs were synthesised based on a published procedure.[10] In detail, 1 mL of the prepared citrate@AuNPs was centrifugated (4000 rpm, 20 min) to yield a pellet that was then redissolved in 1 mL of Tris buffer. Then, a buffered (Tris 10 mM, pH 8.5) solution of dopazide (1mg/mL) was freshly prepared and added to the citrate@AuNP solution whilst stirring. The reaction was performed in this buffer to aid the deprotonation of the hydroxy groups of the dopazide, resulting in displacement of the citrate stabilisers. This solution was then left to react for 16 h at room temperature. The resulting solution was centrifuged (4000 rpm, 10 min, 3x) to remove excess unreacted species and the collected pellet was redissolved in 1 mL of Milli-Q water.

Preparation of the PDEVP@AuNPs *via* click chemistry

To initiate the coupling process, the alkyne PDEVP polymer (4 mg/mL) was prepared in Mili-Q water in a clean glass vial before the addition of the catalyst Cp*RuCl(COD) (0.2 mg/mL), also in Mili-Q water. The solution was then subjected to sonication for 15 min to facilitate the dispersion and interaction of the polymer and catalyst at a molecular level. To facilitate the click reaction of the alkyne with an azide-containing substrate, the TMS group needs to be quantitatively removed with potassium carbonate (K_2CO_3) in methanol (Scheme 1 in the main manuscript) while maintaining the molecular weight and the polydispersity of the polymers (Figures S10 and S15). Next, the dopazide@AuNPs solution from the previous step (1 mL) was added to the vial, sealed and allowed to stir at room temperature for 24 h. The resulting solution was centrifuged (4000 rpm, 10 min, 3x) to remove excess of unreacted species and the collected pellet was redissolved in 1 mL of Milli-Q water and purified by dialysis using cellulose dialysis membranes (Sigma-Aldrich, D9652) with a MWCO of 14 kDa.

To validate the results obtained from the experimental procedure, control experiments were conducted under identical conditions with a PDEVP polymer without terminal alkyne (*Test i*) or without the catalyst (*Test ii*).

4. End-group analysis

To determine the attachment of the alkyne-containing initiator oligomers of PDEVP are prepared as described for the polymerisation with 5 equiv. of DEVP. 10 µL of the reaction mixture are withdrawn after 5 minutes and dissolved in HPLC-grade acetonitrile for subsequent ESI-MS analysis.

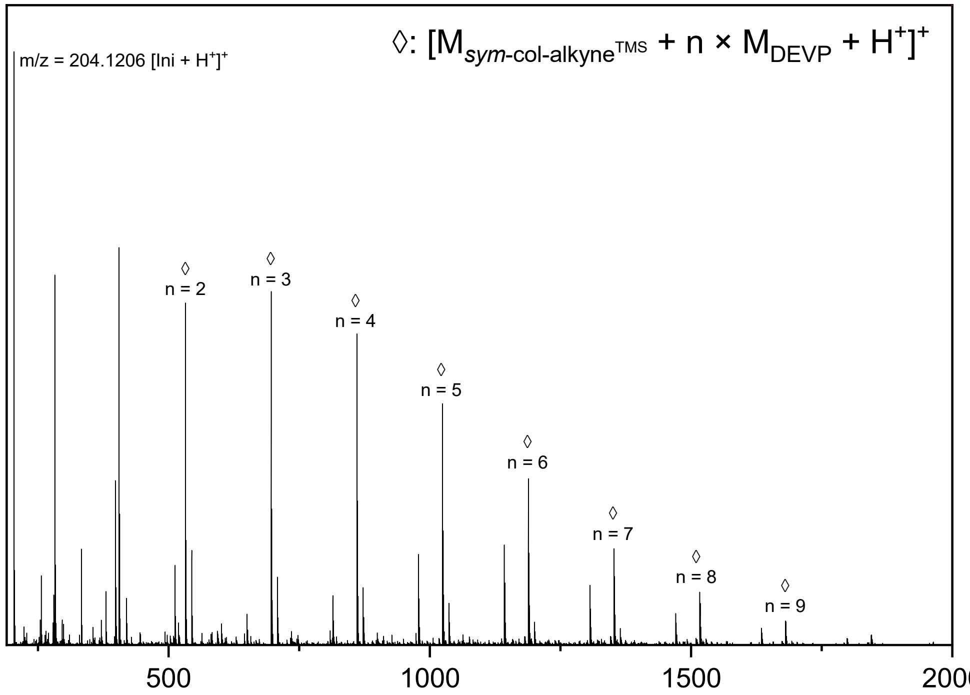

Figure S2: ESI-MS spectrum of short-chain PDEVP prepared with in situ generated Cp_2Y(sym-col$_{alkyne}$) showing a main series corresponding to [n x M_{DEVP} + $M_{sym\text{-}colalkyne}$ + $H^+]^+$ (black) indicating successful attachment of the alkyne sym-collidine derivative to the PDEVP chain.

5. SEC TRACES

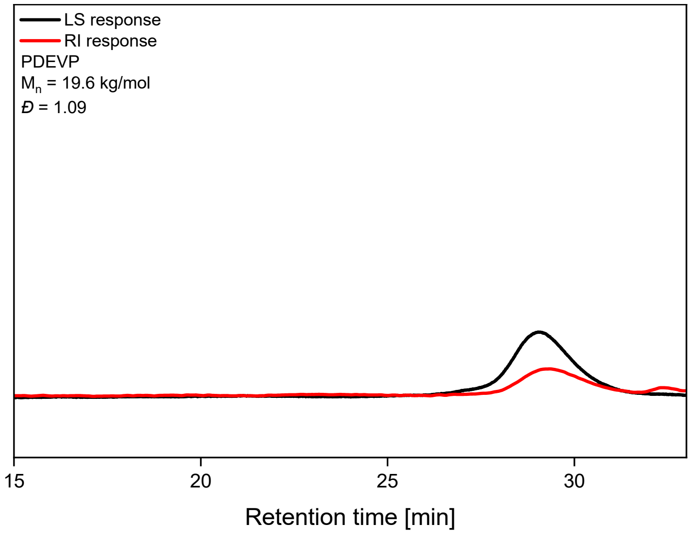

Figure S3: SEC-MALS of PDEVP prepared with Cp_2Y(*sym*-col-CCTMS) (Table S2, entry 1).

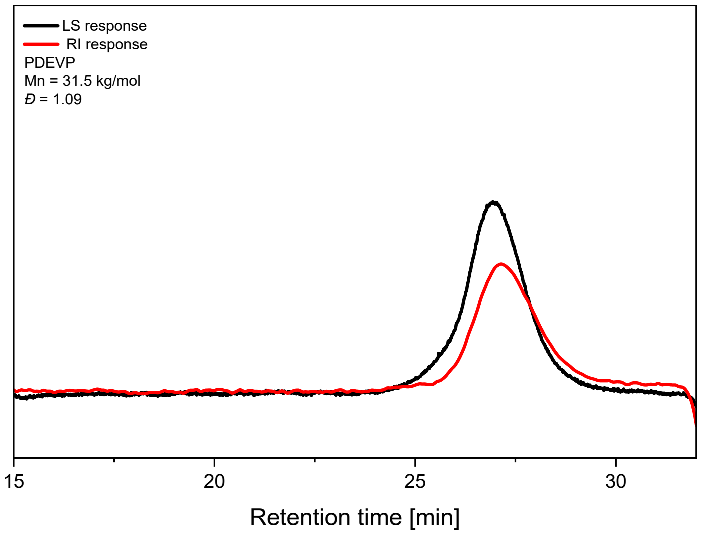

Figure S4: SEC-MALS of PDEVP prepared with Cp_2Y(*sym*-col-CCTMS) (Table S2, entry 2).

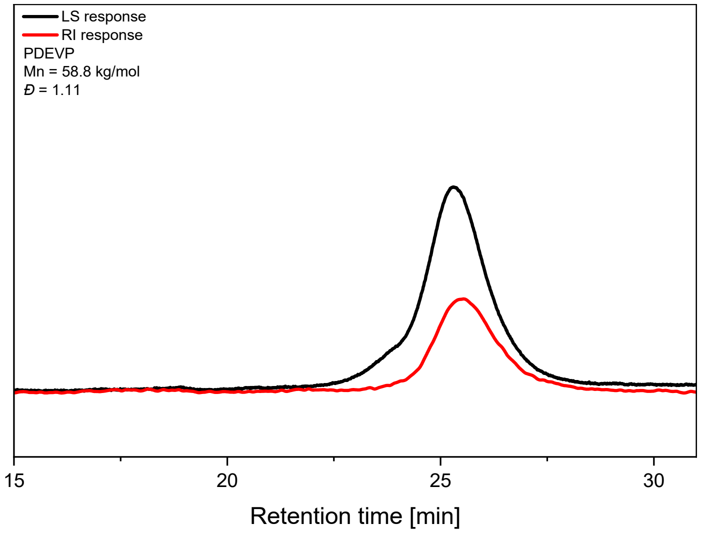

Figure S5: SEC-MALS of PDEVP prepared with Cp_2Y(*sym*-col-CCTMS) (Table S2, entry 3).

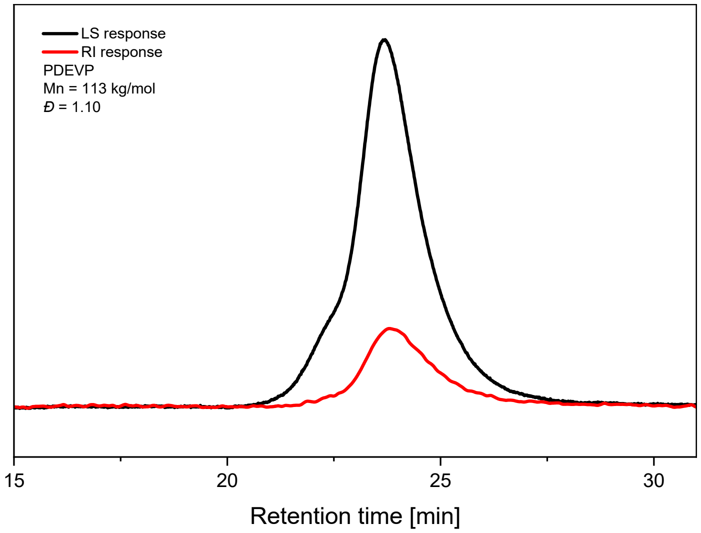

Figure S6: SEC-MALS of PDEVP prepared with Cp_2Y(*sym*-col-CCTMS) (Table S2, entry 4).

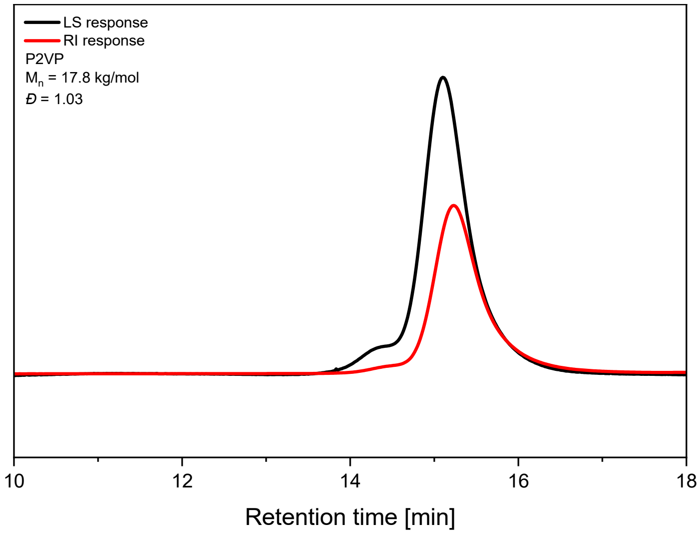

Figure S7: SEC (DMF) of P2VP prepared with Cp_2Y(*sym*-col-CCTMS) (Table S2, entry 5).

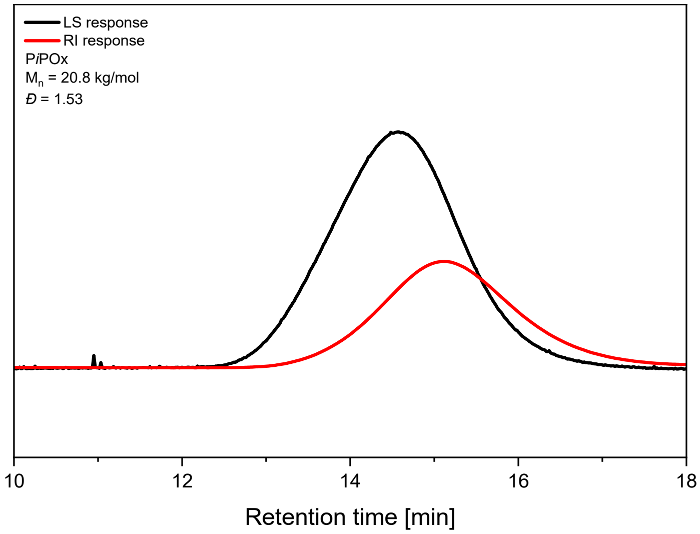

Figure S8: SEC (DMF) of P*i*POx prepared with Cp_2Y(*sym*-col-CCTMS) (Table S2, entry 6).

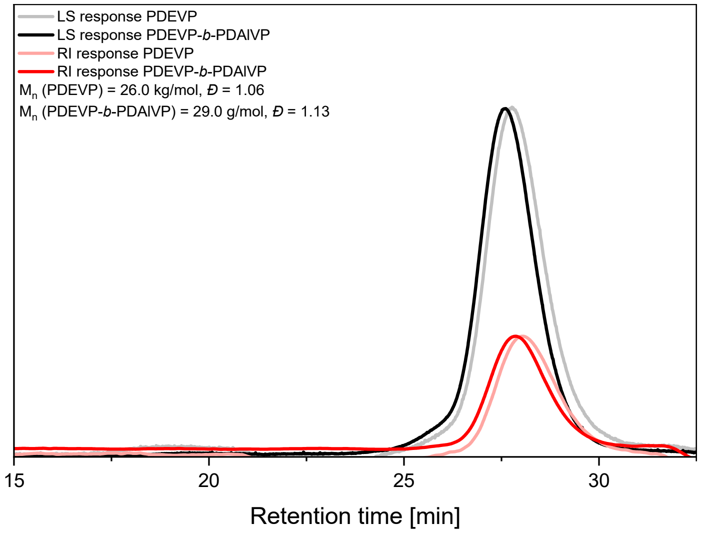

Figure S9: SEC-MALS of PDEVP-*b*-PDAlVP prepared with Cp_2Y(*sym*-col-CCTMS) (Table S2, entry 7).

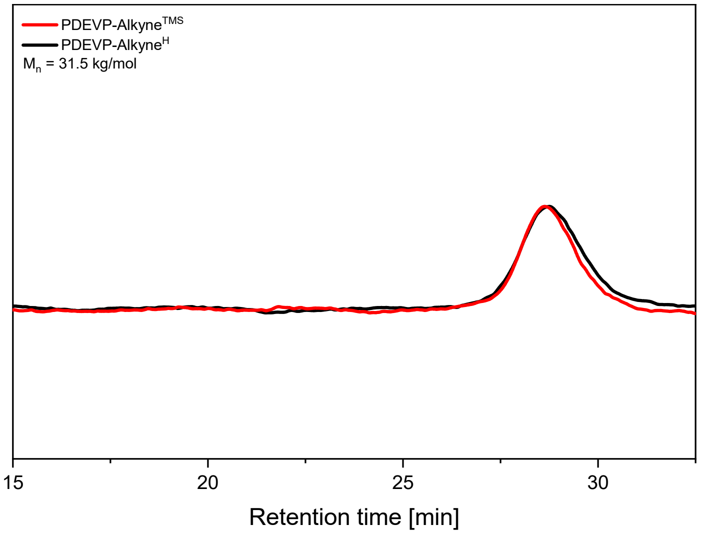

Figure S10: SEC-MALS of TMS-protected PDEVP (red) and deprotected PDEVP (black).

6. NMR data

Group-transfer polymerisation

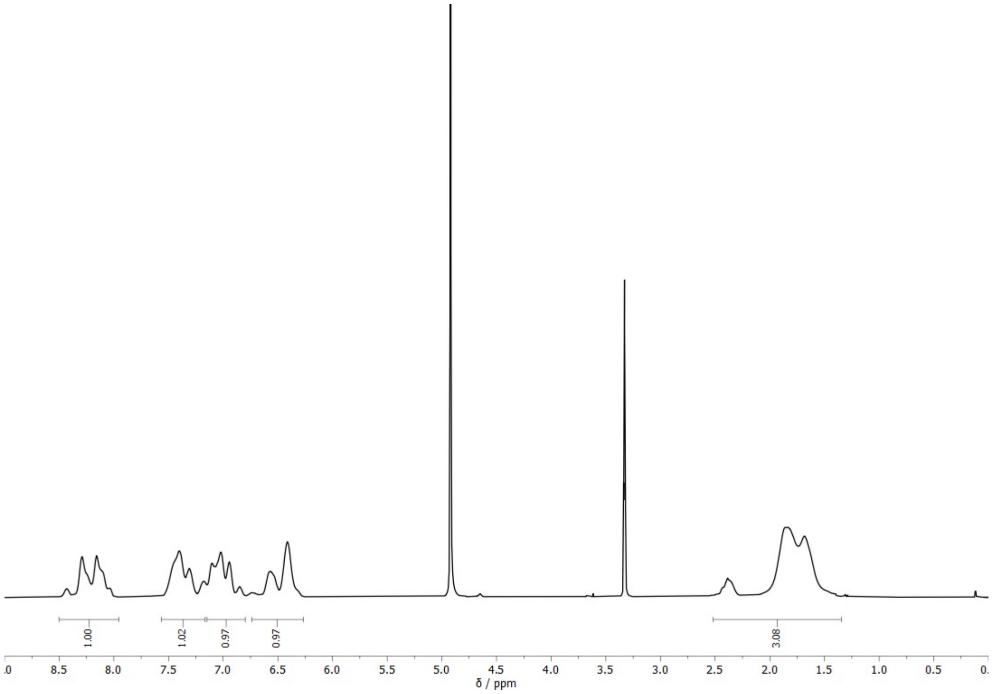

Figure S11: ^{1}H-NMR Spectrum of TMS-protected P2VP in MeOD.

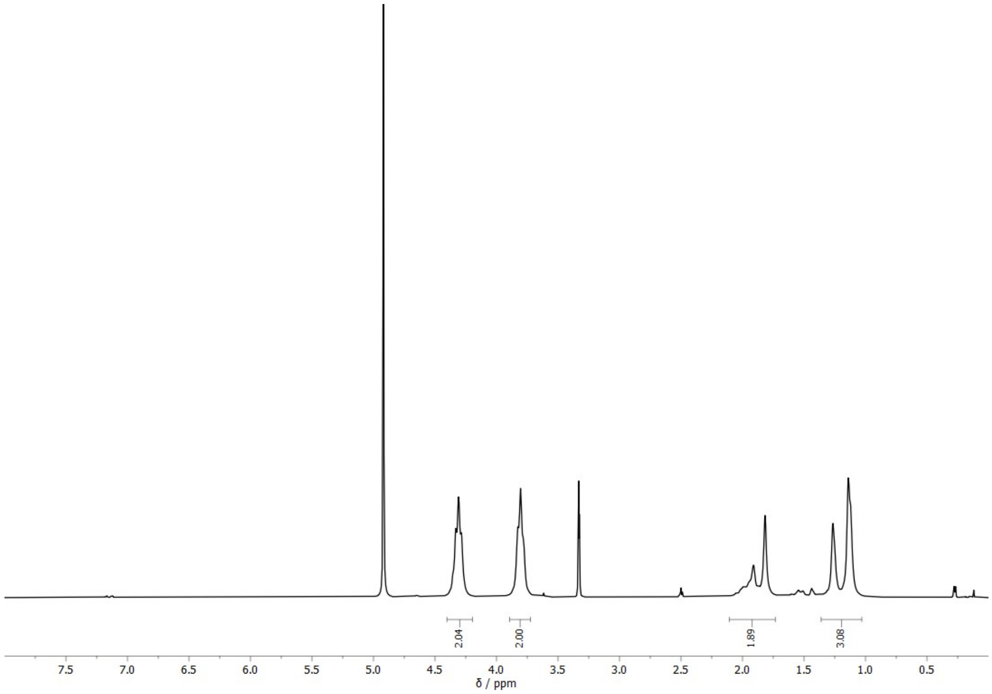

Figure S12: ^{1}H-NMR Spectrum of TMS-protected P*i*POx in MeOD.

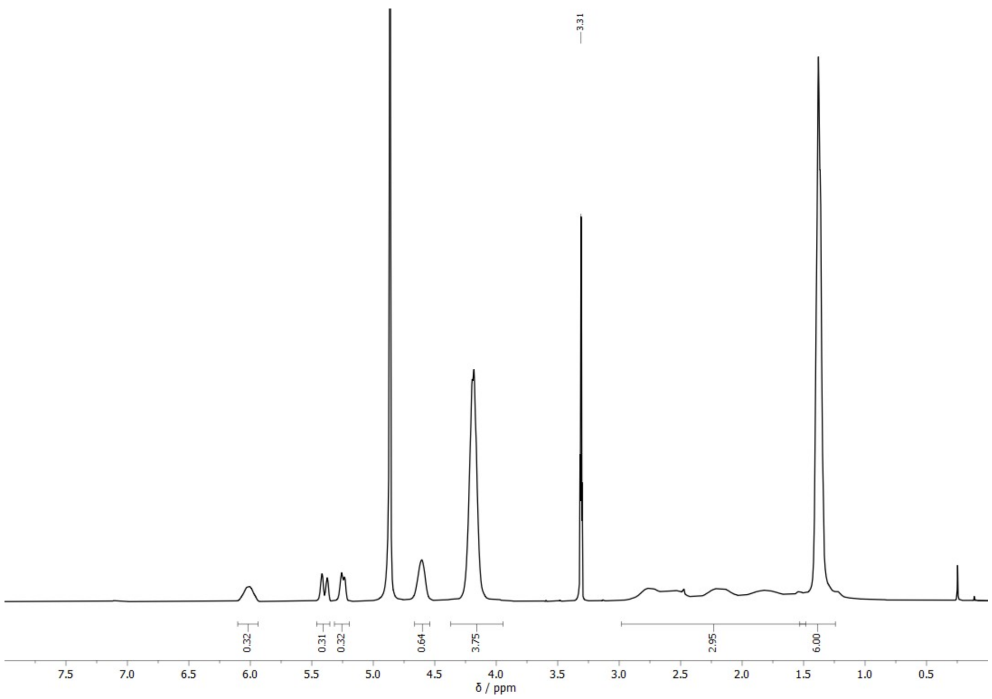

Figure S13: ^{1}H-NMR Spectrum of TMS-protected PDEVP-*b*-PDAlVP block copolymer in MeOD.

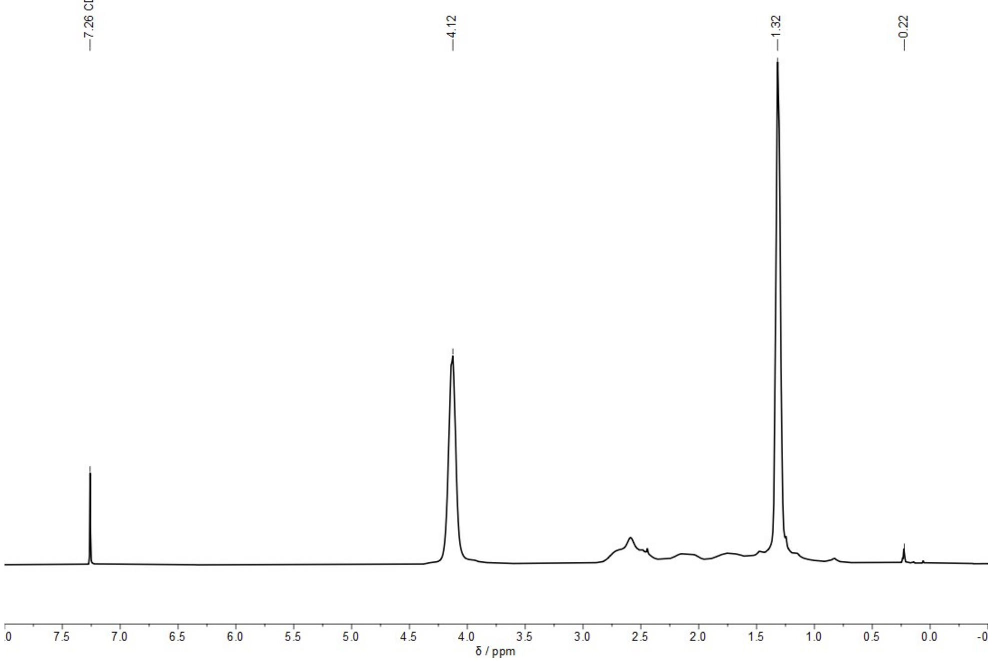

Figure S14: ^{1}H-NMR Spectrum of TMS-protected PDEVP in $CDCl_3$.

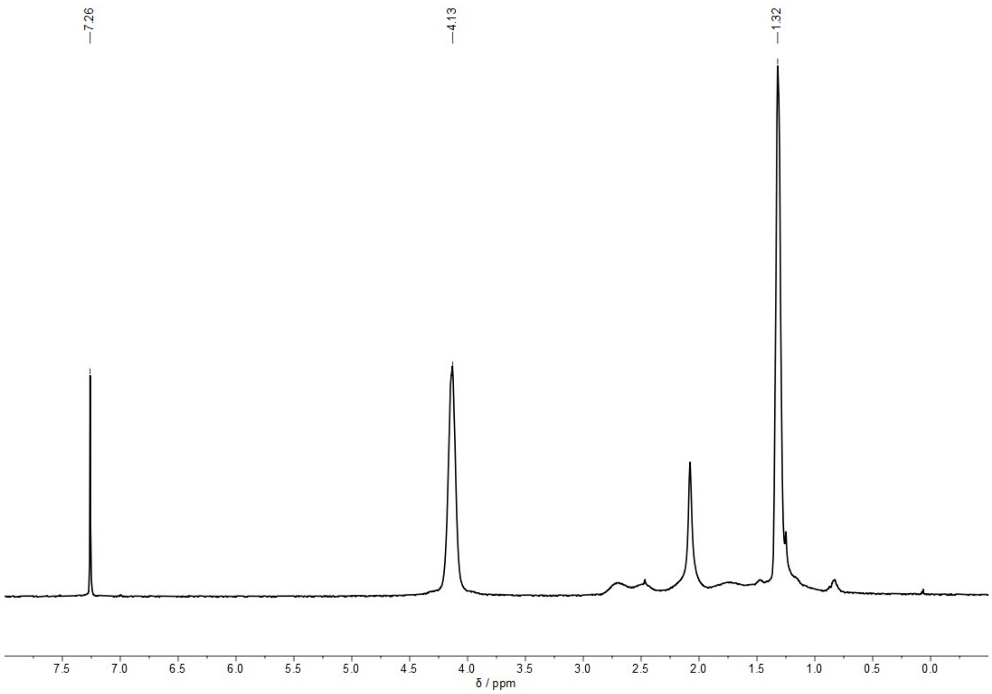

Figure S15: ^{1}H-NMR Spectrum of deprotected PDEVP in $CDCl_3$.

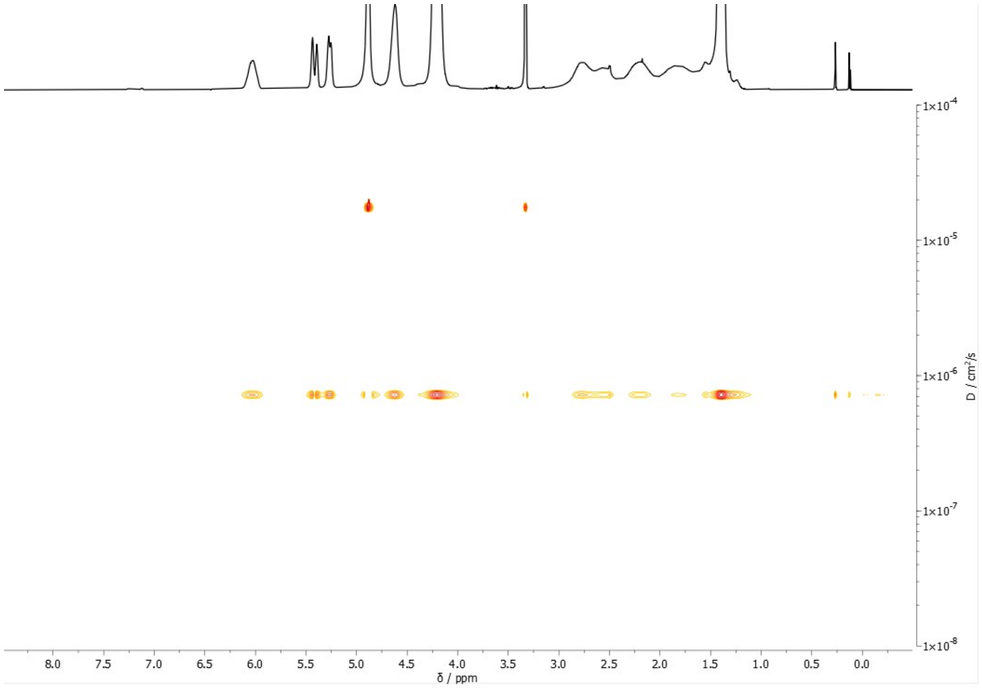

Figure S16: ^{1}H DOSY-NMR Spectrum of TMS-protected PDEVP-*b*-PDAlVP block copolymer in MeOD.

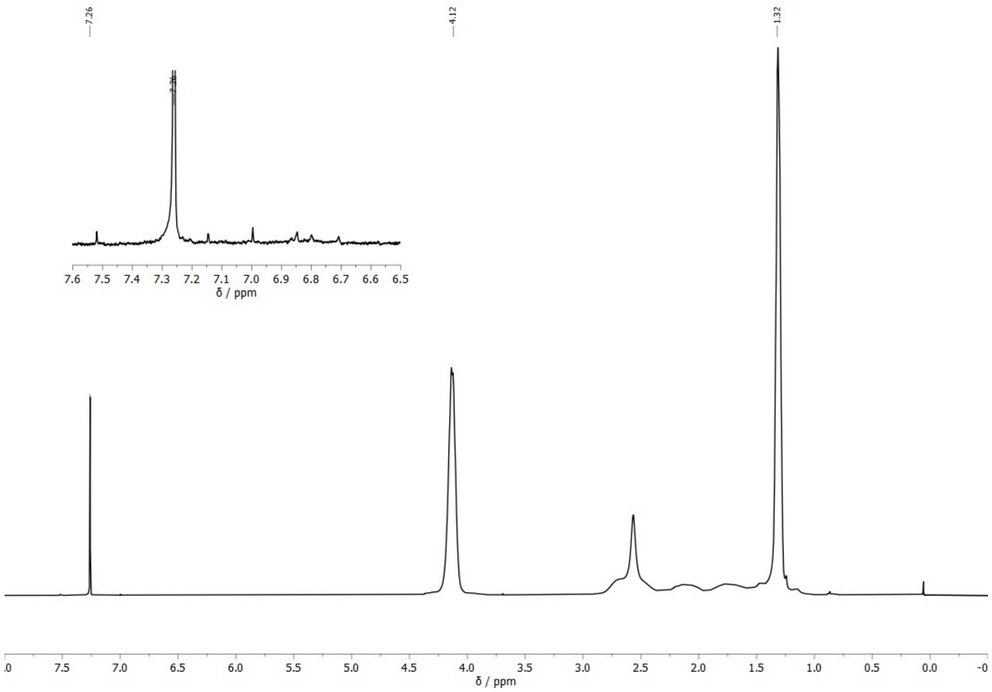

Figure S17: ^{1}H-NMR Spectrum of Coumarine-N_3 functionalised PDEVP in $CDCl_3$.

7. Fluorescence emission

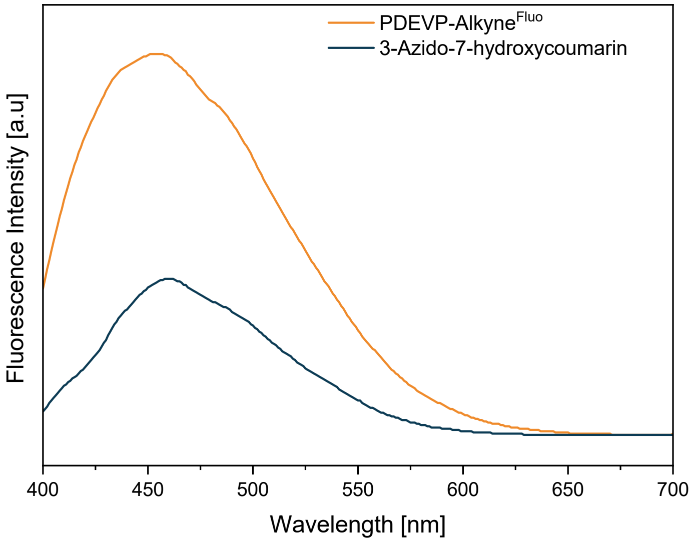

Figure S18: Fluorescence study of coumarine-functionalised PDEVP.

8. AuNP Characterisation

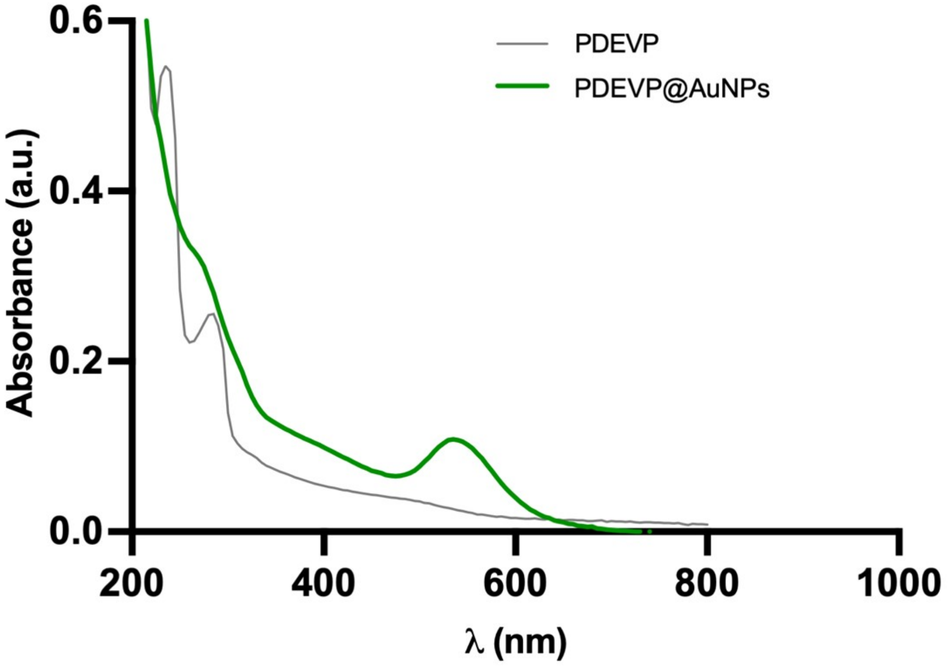

Figure S19: UV-Vis absorption spectra of PDEVP@AuNPs (green) and PDEVP-Alkyne polymer (grey) in Milli-Q water.

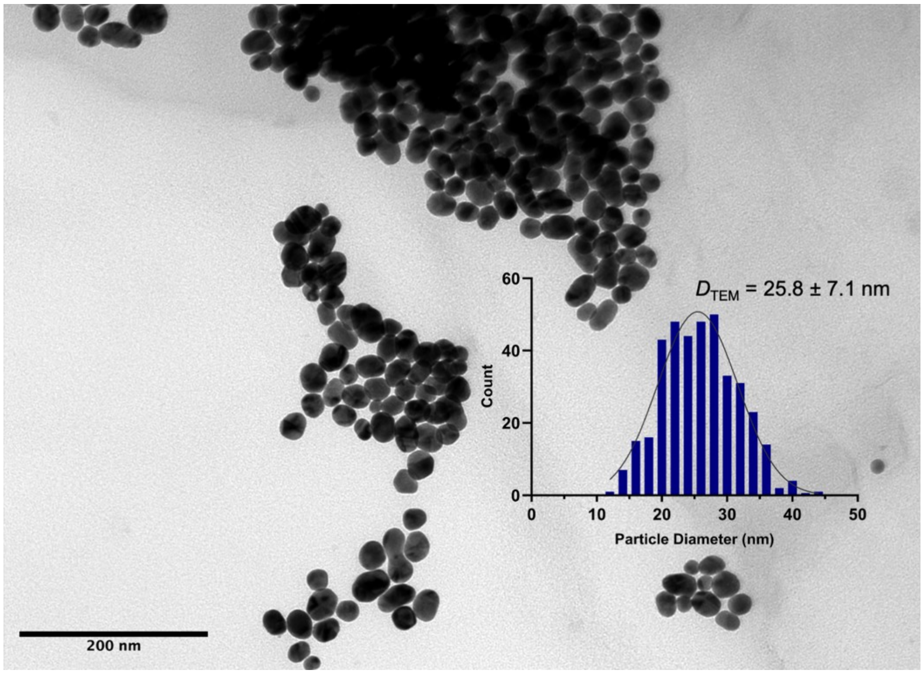

Figure S20: Representative TEM image of citrate@AuNPs (scale bar: 200 nm) with the particle size histogram displaying an average size of 25.8 ± 7.1 nm.

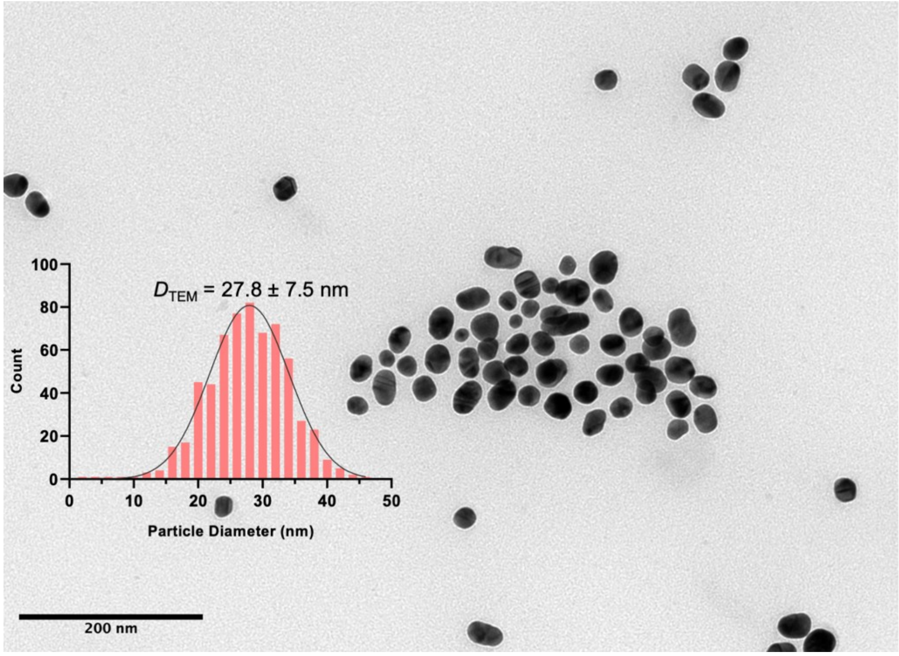

Figure S21: Representative TEM image of dopazide@AuNPs (scale bar: 200 nm) with the particle size histogram displaying an average size of 27.8 ± 7.5 nm.

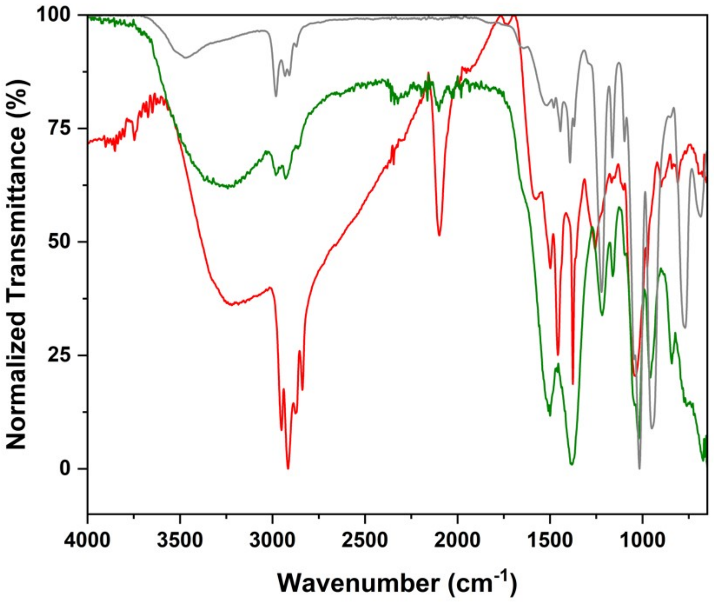

Figure S22: IR Spectra of dopazide@AuNPs (red), PDEVP-Alkyne polymer (grey) and PDEVP@AuNPs (green).

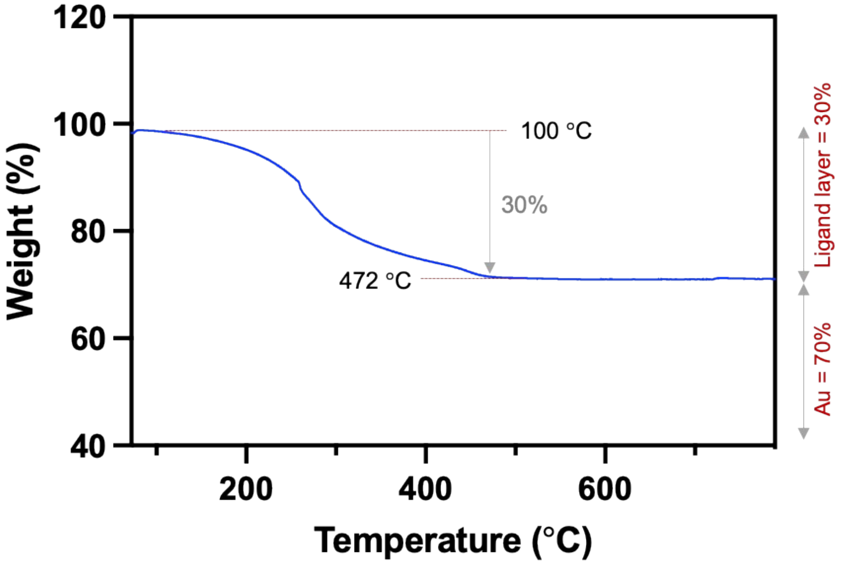

Figure S23: TG curve of PDEVP@AuNPs.

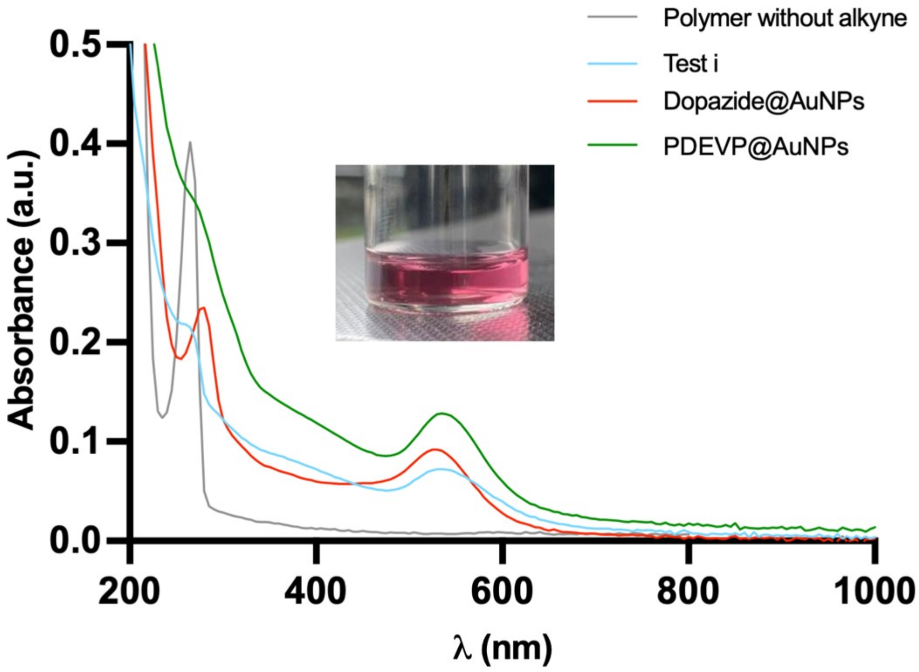

Figure S24: UV-Vis absorption spectra of dopazide@AuNPs (red), PDEVP@AuNPs (green) and test i (light blue) and polymer without alkyne (grey) in Milli-Q water (insert: image of test i NP solution).

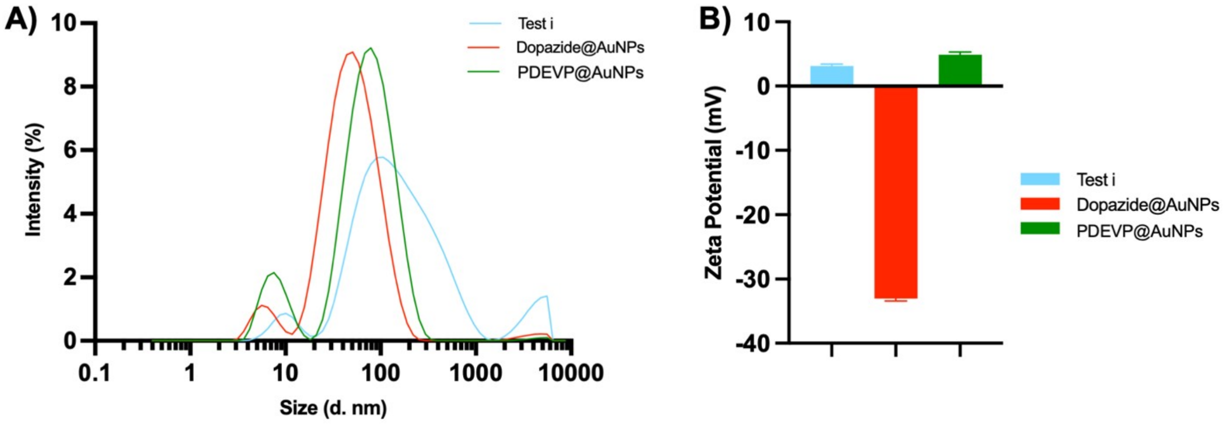

Figure S25: Representative plots of **A**) intensity size distribution by DLS, and **B**) zeta potential of dopazide@AuNPs (red), PDEVP@AuNPs (green) and test i (light blue) in Milli-Q water displaying the average values from 3 repeats.

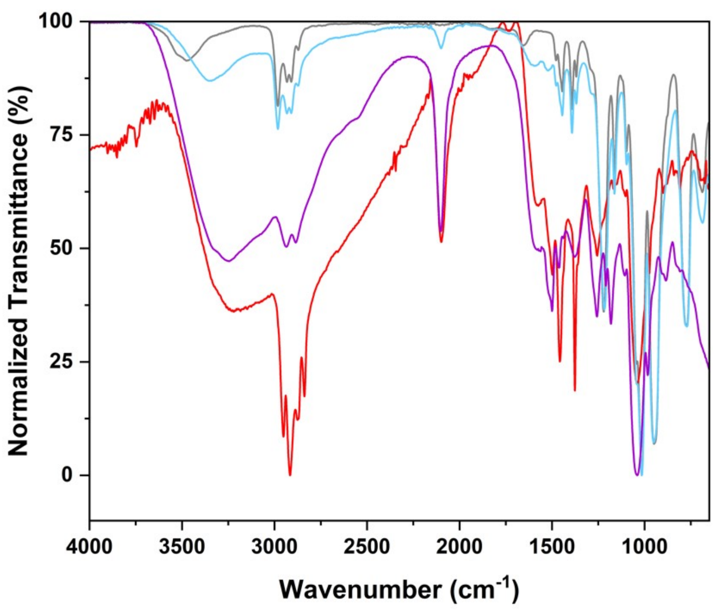

Figure S26: IR Spectra of dopazide@AuNPs (red), PDEVP polymer without alkyne (grey), test i (light blue) and test i supernatant (purple).

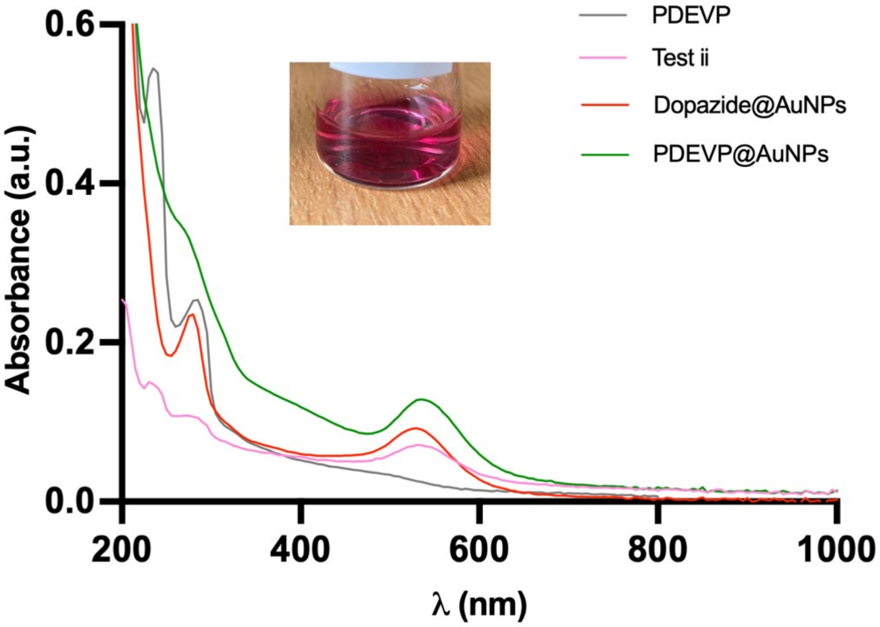

Figure S27: UV-Vis absorption spectra of dopazide@AuNPs (red), PDEVP@AuNPs (green) and test ii (pink) and PDEVP-alkyne polymer (grey) in Milli-Q water (insert: image of test ii NP solution).

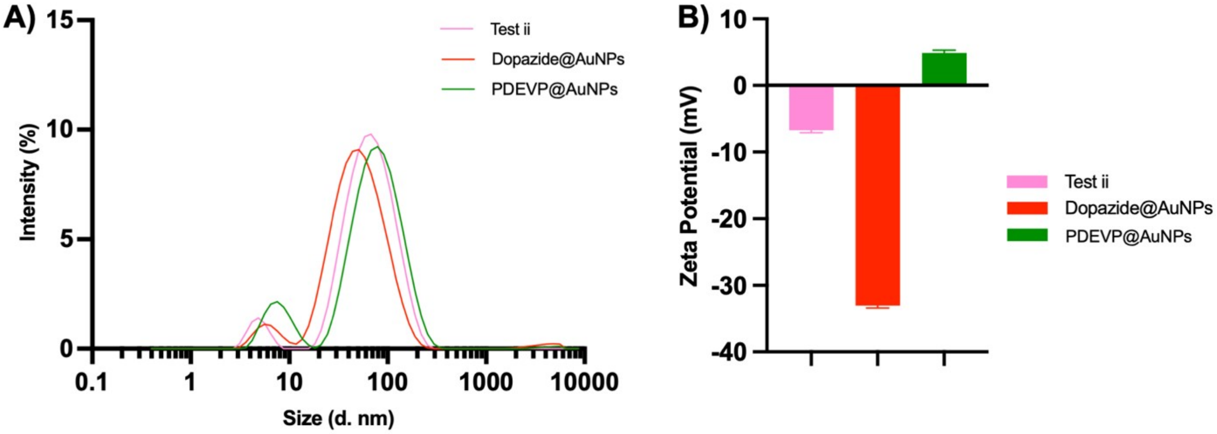

Figure S28: Representative plots of **A**) intensity size distribution by DLS, and **B**) zeta potential of dopazide@AuNPs (red), PDEVP@AuNPs (green) and test ii (pink) in Milli-Q water displaying the average values from 3 repeats.

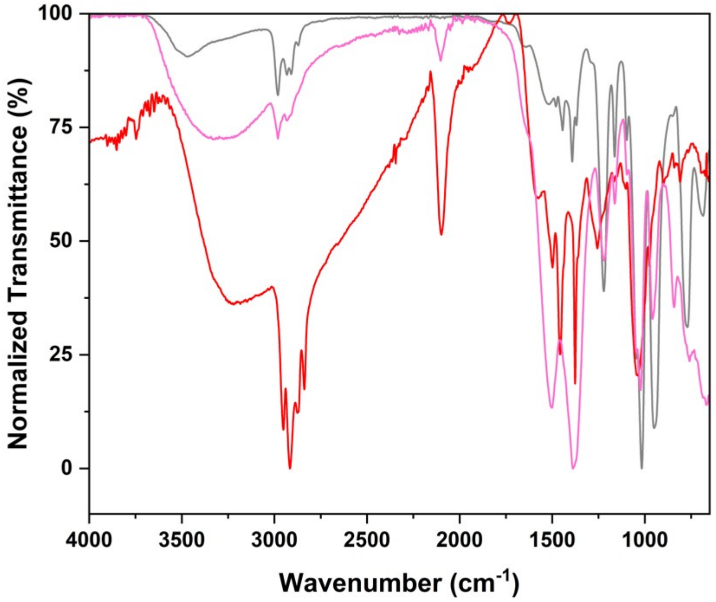

Figure S29: IR Spectra of PDEVP-Alkyne polymer (grey), dopazide@AuNPs (red) and test ii (pink).

References

1 a) X. Zhang, Q. Zhang, C. Xie, A. Gao, Z. Chang, J. Kwon Oh, P. Yang and P. Li, *J. Polym. Sci. Part A: Polym. Chem.*, 2016, **54**, 1396–1408; b) C. Schwarzenböck, P. J. Nelson, R. Huss and B. Rieger, *Nanoscale*, 2018, **10**, 16062–16068;

2 G. D. Vaughn, A. K. Krein and J. A. Gladysz, *Organometallics*, 1986, **5**, 936–942.

3 K. C. Hultzsch, P. Voth, K. Beckerle, T. P. Spaniol and J. Okuda, *Organometallics*, 2000, **19**, 228–243.

4 C. Schwarzenböck, A. Schaffer, P. Pahl, P. J. Nelson, R. Huss and B. Rieger, *Polym. Chem.*, 2018, **9**, 284–290.

5 B. S. Soller, S. Salzinger, C. Jandl, A. Pöthig and B. Rieger, *Organometallics*, 2015, **34**, 2703–2706.

6 P. C. Lee and D. Melsel, *J. Phys. Chem.*, 1982, **86**, 3391–3395.

7 D. Winkelhaus, B. Neumann, H.-G. Stammler and N. W. Mitzel, *Dalton Trans.*, 2012, **41**, 9143–9150.

8 S. Pagoti, D. Dutta and J. Dash, *Adv. Synth. Catal.*, 2013, **355**, 3532–3538.

9 J.-G. Wang, X. Hua, M. Li and Y.-T. Long, *ACS Appl. Mater. Interfaces*, 2017, **9**, 3016–3023.
